# P15 Knowledge mobilization of the new UK-adapted Access, Watch, Reserve and Other classifications

**DOI:** 10.1093/jacamr/dlaf230.022

**Published:** 2025-12-04

**Authors:** Rachel Berry, Bee Yean Ng, Diane Ashiru-Oredope

**Affiliations:** UK Health Security Agency, London, UK; UK Health Security Agency, London, UK; UK Health Security Agency, London, UK; University of Nottingham, Nottingham, UK

## Abstract

**Background:**

Antimicrobial resistance (AMR) remains a critical global health threat. In response, the WHO developed the AWaRe classification in 2017 to support antibiotic stewardship by categorizing antibiotics into three groups: **A**ccess (narrow-spectrum, first-line agents), **Wa**tch (broader-spectrum, second-line agents) and **Re**serve (last-resort options). In 2024, this classification was adapted for the UK context through a Delphi consensus process, with the method published in January 2025 in a peer-reviewed journal and the results on the GOV.UK website. This adaptation supports Target 4b of the UK AMR National Action Plan (NAP): achieving 70% of antibiotic use from the Access category by 2029.

**Objectives:**

To mobilize knowledge of the updated UK-specific AWaRe classification among stakeholders in England using the Knowledge-to-Action (KTA) Framework.

**Methods:**

The KTA framework guided stakeholder identification, message tailoring, awareness-building and engagement through multiple channels. Dissemination activities included publication of the classification, a peer-reviewed article, social media campaigns and direct communication with key stakeholders. Engagement was further supported through meetings, presentations and discussions to explore barriers and implications for implementation.

**Results:**

Initial results indicate strong engagement. The GOV.UK webpage received 1130 unique visits within two months of publication. Social media outreach across Facebook, LinkedIn, Instagram and X generated 84 577 impressions, 3456 engagements (4.1% engagement rate) and 83 shares. Examples of additional mobilization efforts included a press release leading to trade journal articles, updates on the Fingertips platform, presentations to the ESPAUR Oversight Group and dissemination through the Antibiotic Guardian newsletter and PrescQIPP Antimicrobial Stewardship Network. Key barriers identified included the need for clearer messaging for frontline staff and the public, debates around the classification of first-generation cephalosporins and concerns about the impact of increasing methenamine use which is classified as ‘Other’.

**Conclusions:**

This project successfully raised awareness and interest in the updated UK-adapted AWaRe classification as a tool to support antimicrobial stewardship among key stakeholders in England. A critical success factor was the early identification of stakeholders and the use of clear, targeted communication across multiple channels. Tailoring messages to specific audiences proved essential in building engagement and understanding. Ongoing collaboration with stakeholders highlighted several areas requiring further attention, including the need for tailored messaging for frontline staff, patients and the public, as well as clarification around the impact of methenamine in achieving National Action Plan (NAP) targets. The project reinforced that knowledge mobilization is not a one-time effort but a continuous process requiring regular evaluation and adaptation. Future work will focus on sustaining awareness, supporting implementation and monitoring progress toward the human health targets outlined in the UK AMR NAP.

